# Ferritin heavy chain 1 expression is correlated with plasma cell exhaustion and acts as a prognostic correlative marker in hepatocellular carcinoma

**DOI:** 10.3389/fimmu.2026.1908857

**Published:** 2026-07-31

**Authors:** Yingwei Pan, Xiangfei Meng, Weizheng Liu, Hangyu Zhang, Qiang Yu, Yinbiao Cao, Jie Zhao, Weidong Duan, Haowen Tang, Zhanyu Yang

**Affiliations:** 1 Faculty of Hepato-Pancreato-Biliary Surgery, Chinese People’s Liberation Army (PLA) General Hospital, Beijing, China; 2 School of Medicine, Nankai University, Tianjin, China

**Keywords:** effector B cells, *FTH1*, hepatocellular carcinoma, plasma cell exhaustion, single cell sequencing

## Abstract

**Background:**

Immunotherapy has emerged as a breakthrough in cancer treatment; however, the functional status of B lineage cells within the tumor microenvironment (TME) remains poorly characterized.

**Objective:**

This study systematically profiles the heterogeneity and dysfunctional exhausted state of plasma cells in the hepatocellular carcinoma (HCC) TME, and screens molecular signatures correlated with plasma cell exhaustion.

**Methods:**

B cell subtypes were identified from a single-cell RNA sequencing (scRNA-seq) dataset comprising 10 untreated patients with primary HCC. Gene expression profiling was performed using transcriptomic data from liver cancer tissues and paired paracancerous tissues. The abundance and distribution of exhausted plasma cells were quantified by immunohistochemistry and multiplex immunofluorescence staining. Furthermore, we analyzed the correlation between plasma cell-specific exhaustion markers and patient prognosis using the TCGA-LIHC cohort.

**Results:**

*FTH1* exhibited elevated expression in tumor-infiltrating plasma cells, and its expression level was significantly correlated with the exhausted phenotype of tumor-resident plasma cells. Kaplan-Meier survival analysis revealed that patients with high *FTH1* expression had significantly shorter overall survival compared to those with low *FTH1* expression (P = 0.0005). Importantly, we observed a significant positive correlation between *FTH1* (a molecular signature correlated with plasma cell exhaustion) and the canonical T cell exhaustion marker PD-1 in HCC tumor tissues (P = 0.01).

**Conclusion:**

This study systematically profiles the molecular landscape of dysfunctional plasma cells within the HCC TME, confirms a correlation between *FTH1* overexpression and plasma cell exhaustion, and provides correlative evidence to support further research on liver cancer immunotherapy targeting plasma cell immunity.

## 1 Background

The latest global statistics report that hepatocellular carcinoma (HCC) is the sixth most common cancer and the second leading cause of cancer-related deaths worldwide (1). Moreover, the inflammation caused by hepatitis virus infection or alcoholism is speculated to be the main cause of HCC tumorigenesis (2). In the past few years, immunotherapy has greatly promoted the development of tumor therapy. Usually, after being stimulated by an antigen, immune cells proliferate and differentiate rapidly into effector cells and exert their immune function. However, in chronic viral infections or the tumor microenvironment (TME), owing to the continuous stimulation of antigens, the process of acquiring the memory phenotype of immune cells is blocked, and the immune cells gradually lose their effective function, exhibiting a state of non-response called immune exhaustion. Thus, reversing the exhaustion of immune cells has become the focus of immunotherapy for malignant tumors (3). Therefore, the role of immune response in cancer progression or prognosis requires further elucidation. The involvement of B cells in the development and progression of cancer is controversial. Infiltrating B lymphocytes were reported to be present in the tumor tissue, producing an IgG antibody to recognize a certain antigen in tumor tissue and inhibiting the growth of the tumor to a certain extent (4). In addition to producing specific antibodies, B cells are also antigen-presenting cells. *In vivo*, they present foreign proteins, especially low concentrations of antigens, to T cells via the immunoglobulin SmIg present on its surface. Moreover, B cells activated by CD40 can replace dendritic cells as antigen-presenting cells and participate in the tumor immune response. Additionally, several B cell activation genes have been reported to be downregulated in patients with hepatocellular carcinoma (HCC) with poor prognosis, indicating that they have a synergistic effect on tumor progression (5–8). However, differentiated mature B cells often show a state of hypofunction and exhaustion (9). Nonetheless, as a majority of the expression components in tumor tissues tend to have a Breg phenotype, an in-depth study of B cells in the TME could provide a new direction in anti-tumor research. Based on the potential mechanisms through which B cells participate in tumor immunity, it can be speculated that B cells have both positive and negative regulatory effects in tumor immunity.

This study reveals that *FTH1* expression is correlated with the exhausted functional state of tumor-infiltrating plasma cells in the tumor microenvironment (TME) of hepatocellular carcinoma (HCC) through the analysis of single-cell sequencing data. This finding provides a theoretical foundation for exploring B cell-related immunotherapies in HCC treatment. Additionally, we aim to utilize tissue gene chips, TCGA database, and clinical sample data to elucidate the relationship between relevant molecular characteristics and plasma cell exhaustion in the HCC TME, and to assess their prognostic significance. Furthermore, we developed a clinical model based on *FTH1*+ plasma cells to accurately predict the prognosis of HCC patients.

## 2 Results

### 2.1 Identification and annotation of B cell populations in the HCC TME

**Figure 1:**
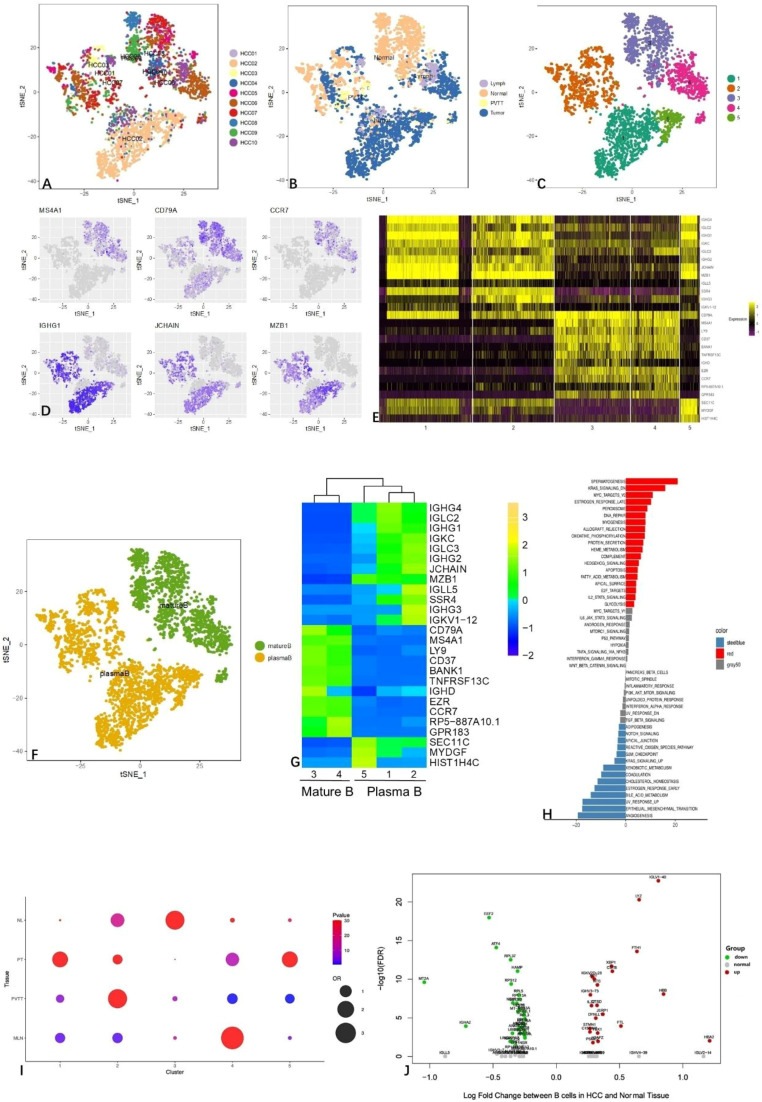
**(A)** Tissue and patient distribution of B cells in the hepatocellular carcinoma (HCC) tumor microenvironment. **(B)** The distribution of these B cells in different tissues. **(C)** The t-SNE distribution map of the five B cell groups. **(D, E)** Heat map of the gene expression of the five B cell population markers. **(F, G)** Subdivision of the five B cell populations into mature B cells and plasma cells. **(H)** The differences of 50 classical signaling pathways between HCC tumor tissues and paracancerous liver tissues. **(I)** Distribution of the B cell population groups. **(J)** Among top upregulated genes in tumor-derived C2 plasma cells versus normal liver plasma cells, *LYZ* exhibited the highest log_2_ fold change (logFC=1.92, P = 2.01 × 10^−25^), followed by *IGLV1-40* (logFC=1.62, P = 7.14 × 10^+28^). *FTH1* was also significantly upregulated with a moderate expression fold change (logFC=0.70, P = 9.99 × 10^−19^).

A total of 3,685 B cells were detected from the single cell atlas of HCC in our previous study. First, the distribution of these B cells among the 10 patients with HCC varied significantly (**Figure 1A**). Second, the distribution of these B cells in different tissues was observed, revealing 1,357 B cells in paracancerous tissues, 1,901 B cells in primary tissues, 178 B cells in portal vein tumor thrombus and 249 B cells in lymph node metastasis (**Figure 1B**). Moreover, 1,033 B cells were observed in the tissues of the HCC-2 patient, which reflected the largest number of B cells observed, while only 11 B cells were found in the tissues of the HCC-1 patient, which was the least number. The 3,685 B cells were clustered unsupervised and divided into five B cell groups, namely 1080 B cells in the C1 group, 938 B cells in the C2 group, 876 B cells in the C3 group, 561 B cells in the C4 group and 230 B cells in the C5 group (**Figure 1C**). Additionally, the C1 and C5 groups mainly consisted of the HCC-2 patient having the largest number of B cells, while the C4 group mainly consisted of the HCC-6 and HCC-10 patients. Therefore, we defined C1, C4 and C5 as specific patient-related B cell groups. Furthermore, C2 and C3 groups were formed by the joint contribution of almost all patients included, hence, we defined them as B cell populations shared by patients.

Then, we identified the marker genes of the above five types of B cell groups (**Figures 1D, E**). Based on the expression of the aforementioned marker genes, the five B cell population groups were further divided into two categories (**Figure 1F**). The C3 and C4 groups highly expressed mature B cell marker genes, namely *CD79A, MS4A1, LY9* and *CD37*, and were thus annotated as mature B cell groups. The C1, C2 and C5 groups highly expressed plasma cell marker genes, namely *IGHG1, IGHG2, IGHG4, JCHAIN* and *MZB1*, and were thus annotated as plasma cell populations (**Figure 1G**). Similarly, mature B cells and plasma cells were enriched in the upper right region and lower left region in the t-SNE map, respectively, with an obvious segmentation but no obvious segmentation within each other.

### 2.2 Significant differences in plasma cell populations between the tumor and paracancerous tissues in patients with HCC

We analysed the distribution preferences of the five B cell populations in the different tissues of HCC. The C3 group of mature B cells was significantly enriched in paracancerous liver tissues whereas plasma cell C1 and C5 groups were significantly enriched in primary HCC tissues. Additionally, the plasma cell C2 group was significantly enriched in the primary foci and portal vein tumor thrombus tissues, while the mature B cell C4 group was significantly enriched in the lymph node metastatic tissues. These results suggest that these B cell populations could have different functions in the occurrence and development of HCC, especially those enriched in the primary and metastatic foci. Considering that this study focused on the common characteristics of the TME of different patients with HCC, we only analysed the C2 and C3 groups. In the C2 and C3 groups, C2 was significantly enriched in tumor tissues, while C3 was observed only in paracancerous liver tissues. Therefore, we focused on the in-depth study of the plasma cell C2 group (**Figure 1I**). Phenotypic profiling of the C2 plasma cell subset revealed negligible expression of*CD19* and *CD20*, low levels of *PRDM1*, and predominant expression of *CD138* and *MZB1* (**Supplementary Figure 1**), indicating that the C2 subset exhibits the canonical molecular signature of mature secretory plasma cells. Furthermore, we found that the HCC tumor tissues infiltrated by C2 group was significantly upregulated in spermatogenesis, KRAS signaling, MYC targets and other pathways but significantly downregulated in angiogenesis, EMT and other pathways (**Figure 1H**). We also analysed the differences in the plasma cell C2 genes and 50 classical signaling pathways between HCC tumor tissues and paracancerous liver tissues. The molecular typing of HCC shows a high degree of heterogeneity, with upregulation of SPERMATOGENESIS, KRAS/MYC pathway representing mechanisms of stem cell maintenance, metabolic reprogramming and immune escape, while the downregulation of angiogenesis and EMT may reflect differentiation status, impaired vascular maturation, or adaptive drug resistance. To resolve the source of SPERMATOGENESIS enrichment in tumor C2 plasma cells, we visualized representative genes from germ cell, iron homeostasis and stress response submodules via dot plot (**Supplementary Figure 5**). Canonical spermatogenic germ cell markers showed negligible expression in both C2 and normal plasma cells, ruling out ectopic germ cell transcriptional activation. In contrast, iron storage genes *FTH1* and *FTL* were specifically upregulated in C2 cells, accompanied by mild elevation of stress-related genes. These data confirm the observed signature enrichment arises from tumor-driven iron and stress programs, rather than germ cell developmental pathways. The upregulation of *LYZ, IGLV1-40, FTH1*, and *CSTB* synergistically enhances B-cell functions by promoting antimicrobial defense (*LYZ*), diversifying antigen recognition (*IGLV1-40*), supporting antibody synthesis via iron homeostasis (*FTH1*), and maintaining cellular integrity through protease inhibition (*CSTB*), whereas the downregulation of *IGHA2* impairs mucosal immune responses by reducing secretory IgA formation (**Figure 1J**). *IGHA2*, as a classical B-cell surface receptor, is highly expressed in activated B cells and plasma cells (24). Its downregulation suggests dysfunction in antibody secretion by plasma cells. We further analyzed the expression profiles of immunosuppressive cytokines *IL-10, IL-35* and *TGF-β* across the five B cell subsets. The C2 plasma cell subset displayed markedly lower cytokine expression levels relative to other B cell populations (**Supplementary Figure 2**). Next, we curated a gene signature consisting of well-established B cell exhaustion markers to calculate an exhaustion module score for each subset. The C2 plasma cell subset yielded higher exhaustion scores compared with all remaining B cell subgroups (**Supplementary Figure 3**). This suggests that the C2 plasma cell group infiltrated by the tumor could be inactivated or depleted. Simultaneously, these results reveal prominent functional differences of C2 plasma cell subsets between tumor and paracancerous tissues. The top three genes upregulated in tumor tissue, ranked by p-value, are *IGLV1-40, LYZ*, and *FTH1*. *IGLV1–40* is a non-coding gene, therefore we do not consider it as a screening target for biomarkers. Lysozyme (*LYZ*) has been observed for the prognostic implications in different types of tumors. Proteomics-based classification has reported that, *LYZ* promoted HCC proliferation and migration in both autocrine and paracrine manners independent of the muramidase activity through the activation of downstream protumoral signaling pathways via cell surface *GRP78*. These results propose *LYZ* as a prognostic biomarker and therapeutic target for the subclass of HCC with an aggressive phenotype (25). However, its expression is predominantly observed in tumor-associated macrophages (TAMs) and neutrophils. Moreover, its relationship with B cells remains understudied, thus rendering it unsuitable as a biomarker. *FTH1* not only exhibits significant differential expression, but evidence from both published studies and our preliminary research demonstrates elevated expression of *FTH1* in tumor-infiltrating immune cells, including CD68^+^ macrophages, CD8^+^ T cells, and B cells (26, 27). To systematically characterize the molecular features of dysfunctional intratumoral C2 plasma cells, we generated three transcriptional heatmaps profiling immune regulators, iron homeostasis mediators and canonical ferroptosis genes across lymph node, paracancerous liver, primary HCC and PVTT tissues. The immune signature heatmap revealed coordinated upregulation of multiple inhibitory checkpoints and immunosuppressive cytokines alongside global downregulation of antigen-presenting molecules in tumor-resident C2 plasma cells, confirming their exhausted immune phenotype (**Supplementary Figure 4A**). Iron metabolism profiling identified *FTH1* as drastically overexpressed exclusively in C2 cells from primary tumors and PVTT rather than non-tumor compartments (**Supplementary Figure 4B**). Ferroptosis transcriptomic analysis further demonstrated that intratumoral C2 plasma cells formed an *FTH1* dominated anti-ferroptosis signature to resist oxidative cell death, while lymph node C2 cells exhibited a pro-ferroptotic profile with minimal *FTH1* expression (**Supplementary Figure 4C**). Collectively, these complementary transcriptional profiles illustrate that C2 plasma cells uniquely undergo coordinated immune exhaustion, *FTH1* centered iron metabolic remodeling and robust anti-ferroptotic reprogramming upon infiltration into HCC lesions, establishing a correlative link between *FTH1* upregulation and the sustained survival of functionally exhausted plasma cells within the immunosuppressive tumor microenvironment. Therefore, we further focused on *FTH1* to analyze its correlation with plasma cell exhaustion phenotype.

### 2.3 FTH1 expression is significantly correlated with plasma cell exhaustion in HCC patients

**Figure 2:**
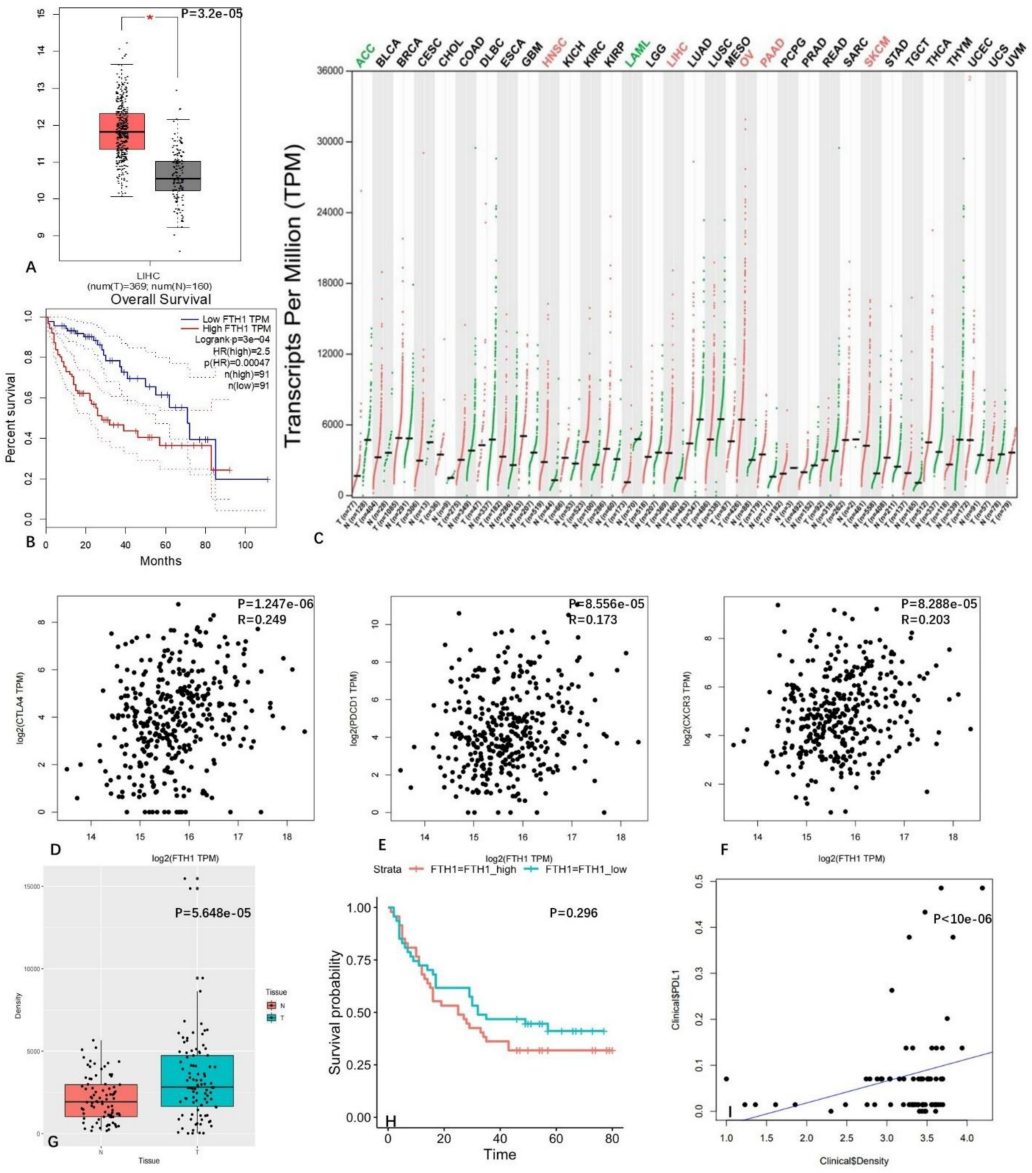
**(A, B)** *FTH1* is highly expressed in hepatocellular carcinoma (HCC) tumor tissues and is closely related to the prognosis of patients. **(C)** Pan-cancer analysis of *FTH1* gene expression. **(D, E)** Correlation between *FTH1* and T cell exhaustion. **(F)** Correlation between *FTH1* and *CXCR3*. **(G, H)** Verification of the correlation between differential expression of *FTH1* and patient prognosis. **(I)** A significant positive correlation between *PD-1* and *FTH1*.

To further verify the results of the single-cell sequencing analysis, we first analysed the difference in *FTH1* gene expression in HCC tumor tissues and paracancerous tissues using 369 patients with HCC obtained from the open database TCGA. The expression of *FTH1* in HCC tumor tissues was significantly higher than that in paracancerous tissues (**Figure 2A**). Then, according to the expression level of *FTH1*, we divided 369 patients with HCC into two groups, the *FTH1* high-expression group and *FTH1* low-expression group and further analysed the difference in overall survival (OS) between the two groups. The results showed that the OS of patients with high *FTH1* expression was significantly shorter than those with low *FTH1* expression (P = 0.00047, **Figure 2B**). Thus, these data demonstrate that *FTH1* overexpression is closely correlated with the exhausted phenotype of tumor-infiltrating plasma cells, and may act as a correlative indicator reflecting plasma cell dysfunction within HCC TME. Additionally, a pan-cancer analysis of the *FTH1* gene was conducted. The differences in *FTH1* expression in different types of tumors and adjacent tissues were also determined. In addition to HCC, the other four types of tumors also revealed a significant upregulation of *FTH1* in tumor tissues, namely Head and Neck squamous cell carcinoma, ovarian cancer (OV), pancreatic adenocarcinoma and Skin Cutaneous Melanoma (SKCM) (**Figure 2C**).

Furthermore, the correlation between *FTH1* and classical exhaustion markers *PD-1* and *CTLA4* expressed on the T cell surface was analysed to validate the reliability of *FTH1* as a molecular marker of plasma cell exhaustion. A significant positive correlation between *FTH1* and T cell exhaustion markers *PD-1* and *CTLA4* was observed in HCC tumor tissues and the correlation P values were 1.247e-06 and 8.556e-05 respectively (**Figures 2D, E**). Concurrently, we also used the collection of plasma cell markers *CXCR3* (28) to score TCGA-bulk-RNA-seq data. Finally, the correlation between *FTH1* gene expression level and *CXCR3* was analysed. The results revealed a significant positive correlation between the expression of *FTH1* and plasma cells in tumor tissues (**Figure 2F**). These results demonstrate that *FTH1* (correlated with plasma cell exhaustion) positively correlates with T cell exhaustion markers *PD-1* and *CTLA4*, suggesting coordinated immune exhaustion of both plasma cells and T cells in HCC TME.

### 2.4 Correlation between FTH1 expression in tumor-infiltrating plasma cells and clinical prognosis in an independent tissue microarray cohort

Consistent with transcriptional data from the TCGA-LIHC cohort, transcriptomic detection on an independent HCC tissue microarray confirmed significantly higher *FTH1* gene expression in tumor tissues than matched peritumoral tissues (**Figure 2G**). Patients with high tumoral *FTH1* transcription had numerically shorter overall survival than those with low *FTH1* expression, though this trend failed to reach statistical significance (**Figure 2H**), which may be attributed to long-term sample storage affecting RNA quality. We further analyzed the correlation between *FTH1* transcript levels and *PD-1* expression in this independent cohort, and a weak but statistically significant positive correlation was observed (R = 0.0669, P = 0.0271; **Figure 2I**).

### 2.5 Tissue microarray validation of the correlation between FTH1 expression and plasma cell exhaustion phenotype

**Figure 3:**
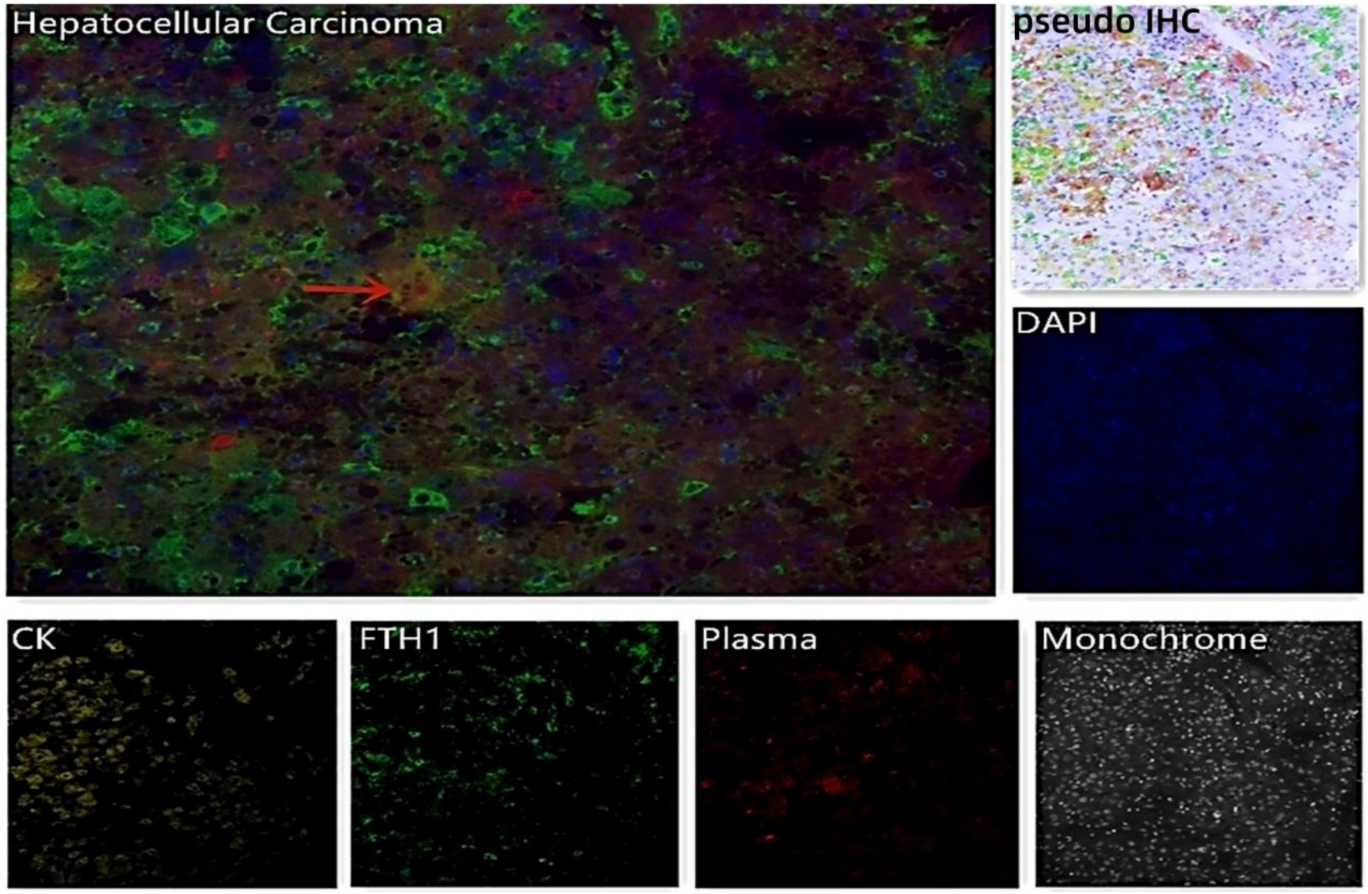
Trichromatic immunofluorescence assay of *FTH1*, Plasma and CK (the red arrow indicates the double positive plasma cells of *FTH1* and Plasma antibodies).

To further verify the association between *FTH1* and the exhausted phenotype of tumor-infiltrating plasma cells in HCC, we conducted multiplex immunofluorescence staining on tissue microarrays from an independent cohort of 90 HCC patients with complete clinical prognostic data. A total of 180 paired tissue samples (90 tumor tissues and 90 matched paracancerous tissues) were co-stained with epithelial marker *CK*, *FTH1*, and specific plasma cell markers. We observed a significant enrichment of *FTH1* positive plasma cells within tumor lesions (**Figure 3**).

### 2.6 Prognostic value of FTH1+ plasma cell abundance in hepatocellular carcinoma

**Figure 4:**
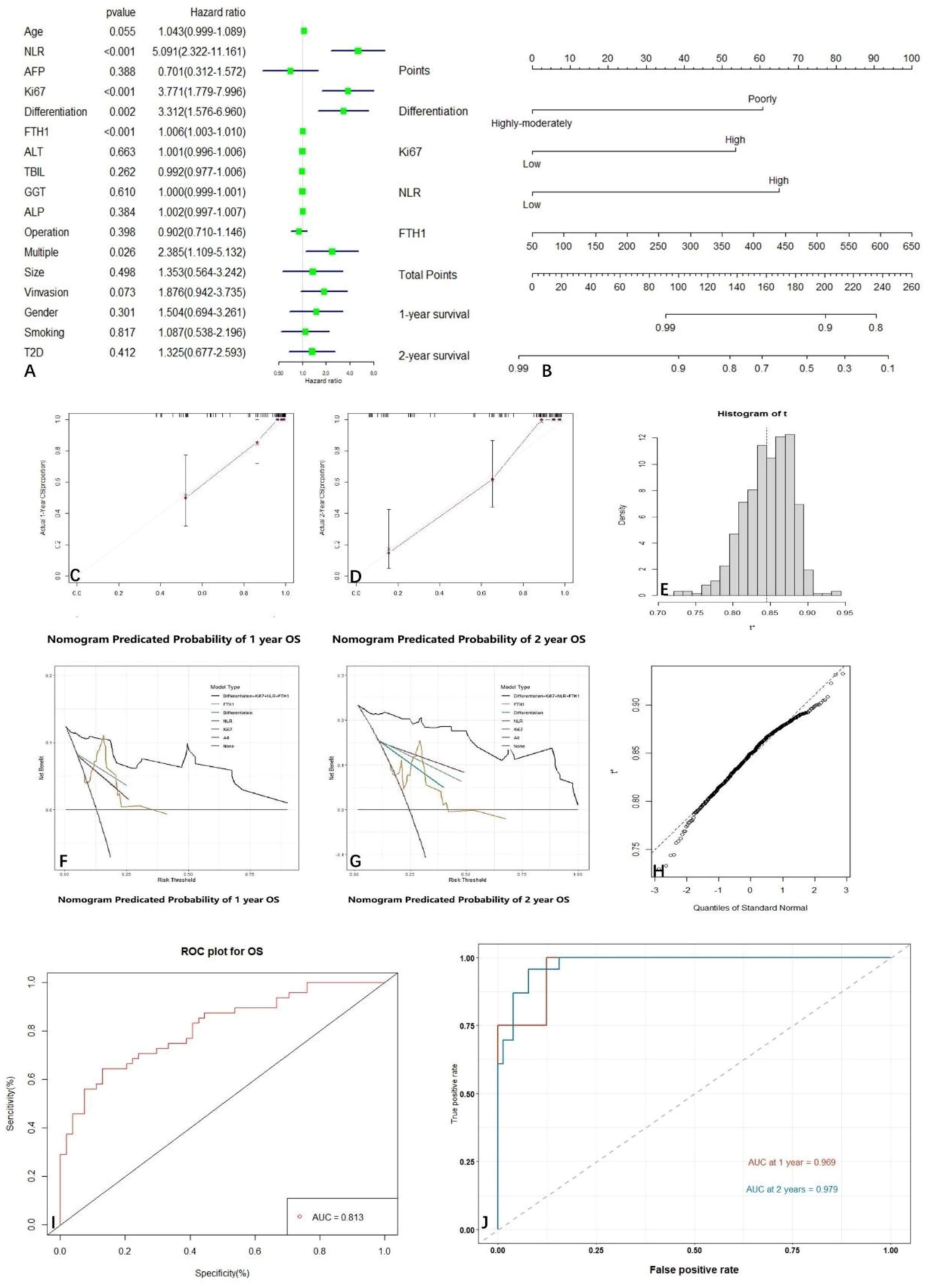
**(A)** Single/multifactorial analysis of postoperative long-term survival in patients with hepatocellular carcinoma. **(B)** The nomogram for predicting the probability of overall survival in HCC patients. **(C, D)** The calibration plots for 1-year and 2-year OS. **(E, H)** The C-index curves for liver cancer patients. **(F, G)** 1-year-2-year OS decision curve analysis for patients with hepatocellular carcinoma. **(I, J)** Area under the curve (AUC) being 0.969 and 0.979 for the entire cohort of liver cancer patients.

Univariate and multivariate Cox proportional hazards regression analyses were performed on our independent cohort of 102 HCC patients who underwent curative surgical resection at our center, with corresponding outcomes summarized in **Tables 1**, **2**. Multivariate Cox regression (**Table 2**) identified NLR, *FTH1* expression, Ki-67 index, and tumor differentiation as independent risk factors linked to postoperative long-term overall survival. Forest plot visualization (**Figure 4**) demonstrated that lower NLR (<1.9), lower Ki-67 index (<20%), reduced *FTH1* expression, and superior tumor differentiation correlated with more favorable long-term survival outcomes, which could act as candidate prognostic indicators. Based on overall survival as the primary clinical endpoint, we developed a predictive nomogram integrating four variables: NLR, *FTH1* expression, Ki-67 index, and tumor differentiation. Within this OS prediction nomogram, higher point scores corresponding to poorer tumor differentiation, elevated Ki-67 index, increased NLR, and stronger *FTH1* expression were associated with worse predicted survival probability for HCC patients in this surgical cohort.

**Table 1:** Univariate analysis of postoperative long-term survival in patients with hepatocellular carcinoma.

Characteristics	Category	HR	HR.95L	HR.95H	P value
Age(y), Mean ± SD	56.1 ± 9.3	1.033	1.001	1.066	0.044^*^
NLR	>1.9/<1.9	5.826	3.171	10.706	<0.001^***^
AFP(ng/ml)	>400/<400	2.603	1.461	4.637	0.001^**^
Ki67(%)	>20/<20	4.466	2.380	8.381	0.000^***^
Differentiation	Poor/Highly-moderately	5.756	3.167	10.460	0.000^***^
FTH1, Mean ± SD	228.4 ± 85.2	1.004	1.001	1.006	0.003^**^
ALT, Median (IQR)	32.5(36.9)	1.003	0.999	1.007	0.089
TBIL(umol/L), Median (IQR)	12.4(7.5)	1.010	0.999	1.022	0.075
GGT(U/L), Median (IQR)	49.5(69.1)	1.000	1.000	1.001	0.585
ALP(U/L), Median (IQR)	82.7(39.7)	1.003	1.000	1.006	0.089
Operation(h), Mean ± SD	4.1 ± 1.4	0.974	0.798	1.189	0.798
Multiple	Multiple/Single	0.987	0.500	1.946	0.969
Size(cm)	>3/<3	1.676	0.834	3.368	0.147
Vein invasion	Yes/No	3.330	1.802	6.152	<0.001^***^
Gender	Male/Female	1.487	0.786	2.811	0.222
Smoking	Yes/No	1.570	0.890	2.769	0.119
T2D	Yes/No	1.178	0.669	2.077	0.571

*P < 0.05, **P < 0.01, ***P < 0.001.

**Table 2:** Multifactorial analysis of postoperative long-term survival of patients with hepatocellular carcinoma.

Characteristics	Category	HR	HR.95L	HR.95H	pvalue
Age(y), Mean ± SD	56.1 ± 9.3	1.041	0.997	1.086	0.065
NLR	>1.9/<1.9	5.503	2.521	12.014	<0.001^***^
AFP(ng/ml)	>400/<400	0.622	0.281	1.375	0.241
Ki67(%)	>20/<20	3.753	1.775	7.937	0.001^**^
Differentiation	Poor/Highly-moderately	3.655	1.727	7.738	0.001^**^
FTH1, Mean ± SD	228.4 ± 85.2	1.007	1.003	1.011	<0.001^***^
ALT, Median (IQR)	32.5(36.9)	1.001	0.996	1.005	0.757
TBIL(umol/L), Median (IQR)	12.4(7.5)	0.991	0.977	1.006	0.249
GGT(U/L), Median (IQR)	49.5(69.1)	1.000	0.999	1.001	0.575
ALP(U/L), Median (IQR)	82.7(39.7)	1.002	0.997	1.007	0.383
Operation(h), Mean ± SD	4.1 ± 1.4	0.910	0.716	1.155	0.438
Multiple	Multiple/Single	2.081	0.989	4.379	0.053
Size(cm)	>3/<3	1.287	0.535	3.100	0.573
Vein invasion	Yes/No	1.916	0.960	3.823	0.065
Gender	Male/Female	1.406	0.652	3.032	0.385
Smoking	Yes/No	1.095	0.542	2.209	0.801
T2D	Yes/No	1.340	0.683	2.631	0.395

*P < 0.05, **P < 0.01, ***P < 0.001.

## 3 Discussion

Hepatocellular carcinoma (HCC) ranks among the most prevalent gastrointestinal malignancies worldwide. Its global incidence and mortality rates continue to rise steadily, with over half of all new cases occurring in China (1). Most patients are diagnosed at intermediate or advanced disease stages, and merely 30% are eligible for curative surgical resection. Radical resection represents the gold-standard treatment for early HCC, yet long-term survival outcomes remain unsatisfactory. Reported 5- and 10-year overall survival (OS) rates reach only 34.4% and 10.5%, respectively, while corresponding disease-free survival (DFS) rates stand at 23.3% and 7.8% (10).

Earlier investigations into crosstalk between HCC and tumor-infiltrating immune cells predominantly focused on T lymphocytes. Accumulating recent evidence, however, highlights the critical involvement of B cells in HCC initiation and progression. Single-cell profiling of the HCC tumor microenvironment (TME) has verified B cell infiltration across all disease stages, with substantial shifts in B cell abundance and functionality linked to distinct tumor histological and molecular subtypes. Given their dual roles in mediating humoral immunity and antigen presentation to orchestrate cellular immune responses, dissecting B cell biology offers promising directions for novel HCC immunotherapies (11, 12). In malignant lesions, self-reactive and pathogen-experienced B cells secrete diverse cytokines to exert either tumor-suppressive or protumor effects (13). Song et al. further demonstrated that regulatory B cells (Breg’s) disrupt dendritic cell function and expand Foxp3^+^ regulatory T cells (Tregs), ultimately accelerating HCC progression (14).

In the present study, we isolated a total of 3,685 B cells from published HCC single-cell transcriptomic datasets and observed striking interpatient heterogeneity in B cell infiltration magnitude. Patient HCC-2 harbored the largest B cell population (1,033 cells), whereas Patient HCC-1 displayed the fewest infiltrating B cells (11 cells). Beyond technical bias during single-cell library construction, profound intrinsic heterogeneity of the HCC TME largely accounts for this variability. Unsupervised clustering partitioned all B cells into five distinct subsets (C1–C5). Clusters C2 and C3 were recognized as pan-patient shared B cell populations, and we systematically characterized tissue-specific distribution preferences across the five subsets. Comparative differential gene expression and canonical signaling pathway enrichment analyses between tumor and paired peritumoral liver tissues identified robust upregulation of *FTH1* within the C2 plasma cell cluster, linking *FTH1* expression to plasma cell hypofunction and exhaustion. Collectively, these data indicate elevated tumoral *FTH1* expression primarily within tumor-infiltrating plasma cells can act as a correlative molecular signature reflecting exhausted plasma cell phenotypes in HCC.

*FTH1* encodes ferritin heavy chain, which possesses ferroxidase activity capable of oxidizing ferrous iron (Fe^2+^) to ferric iron (Fe^3+^). Nuclear ferritin formed exclusively by H-type subunits remains incompletely characterized, yet existing data implicate *FTH1* in shielding genomic DNA from oxidative damage and modulating transcriptional programmed to drive tumor progression (15). Mechanistically, *FTH1* modulates inflammation-associated angiogenesis by binding high-molecular-weight kininogen (HKa) and abrogating its endothelial anti-angiogenic activity *in vitro* and *in vivo* (16, 17). *FTH1* also interacts with multiple oncogenic signaling mediators, including *CXCR4*, a chemokine receptor broadly overexpressed across human solid malignancies (18). Under oxidative stress, *FTH1* stabilizes and activates the tumor suppressor p53 (19). Prior work has further documented protumor roles for *FTH1* across multiple cancer types: it promotes malignant progression in non-small cell lung carcinoma, breast cancer and ovarian cancer, and drives metastatic capacity in melanoma (20–23).

Our multi-omics and tissue staining analyses further validated *FTH1* as a correlative signature indicative of exhausted plasma cell states in HCC. Bulk and tissue-level transcriptomic profiling revealed a statistically significant positive correlation between tumoral *FTH1* abundance and the expression of canonical T cell exhaustion markers. Physiologically, functional B cells execute anti-tumor immunity via three core mechanisms: secreting tumor-targeting antibodies to elicit humoral responses, serving as professional antigen-presenting cells to boost T cell-mediated cytotoxicity, and releasing pro-inflammatory cytokines to sustain T cell activation. Accordingly, characterizing the molecular features of exhausted plasma cells and their clinical correlation with HCC proliferation and progression is vital to inform strategies for improving long-term patient survival.

Multivariate Cox regression analysis based on our independent surgical cohort of 102 HCC patients identified *FTH1*-high plasma cells as an independent adverse prognostic factor for postoperative survival. Patients with low *FTH1*-expressing plasma cells exhibited markedly prolonged median OS relative to those with abundant *FTH1*-high plasma cells. Consistent with prior clinical reports, elevated Ki-67 index, poor tumor differentiation and elevated NLR (>1.9) constituted independent risk factors for postoperative tumor recurrence. To facilitate individualized survival prediction, we constructed an OS nomogram incorporating four clinically accessible variables: NLR, tumoral *FTH1* expression, Ki-67 index and tumor differentiation grade. C-index and time-dependent AUC metrics demonstrated robust predictive performance of this integrated nomogram within our single-center cohort, outperforming any single prognostic variable alone and supporting the predictive value of *FTH1*-expressing plasma cells for HCC patient outcomes.

We hypothesized that B cells must retain intact mature effector functionality to exert anti-tumor cytotoxicity and antibody secretion. Within the immunosuppressive HCC TME, tumor-resident plasma cells frequently acquire a hypofunctional, exhausted phenotype. Deep mechanistic research into plasma cell biology may therefore unlock novel B cell-targeted immunotherapeutic approaches for HCC. Our data additionally confirmed a positive correlation between *FTH1* levels and *PD-1* (a well-established T cell exhaustion marker) in HCC lesions.

Notably, all transcriptomic and multiplex immunofluorescence analyses in this study only reveal statistical correlations between upregulated *FTH1* expression and exhausted plasma cell phenotypes in the HCC tumor microenvironment. Further *in vitro* functional experiments and *in vivo* genetic intervention models are needed to clarify the causal regulatory pathway connecting *FTH1* to plasma cell hypofunction. Another notable limitation stems from the survival analyses based on TCGA bulk RNA-seq data. Bulk transcriptomic profiles integrate gene expression signals from all cell types within tumor tissues, including malignant hepatocytes, macrophages and multiple immune subsets. Consequently, the observed prognostic correlation between *FTH1* and overall survival cannot be exclusively attributed to plasma cell-intrinsic *FTH1* expression. Future independent cohort analyses using cell-specific spatial quantification are required to precisely clarify the plasma cell-specific prognostic impact of *FTH1*.

## 4 Conclusion

This study describes the heterogeneous distribution of plasma cell subsets across HCC patients, which is largely attributed to the high heterogeneity of the HCC tumor microenvironment. Transcriptomic and histological data consistently support a strong statistical correlation between *FTH1* upregulation and plasma cell exhausted phenotype in HCC lesions. Additionally, *FTH1* expression positively correlates with the abundance of exhausted T cells within tumor tissues, revealing coordinated immune dysfunction of plasma cells and T cells in HCC. Our current data only support correlative associations instead of causal effects between *FTH1* and plasma cell exhaustion. This correlative molecular evidence provides novel insights for developing plasma cell-targeted immunotherapeutic strategies for HCC, and further functional researches are warranted to dissect the causal regulatory mechanism of *FTH1* in plasma cell immune dysfunction.

## 5 Materials and methods

### 5.1 Single-cell RNA library construction and sequencing

FASTQ files of HCC single-cell RNA-seq data were obtained from our previous study (Lu, Yiming et al. “A single-cell atlas of the multicellular ecosystem of primary and metastatic hepatocellular.” Nature communications vol. 13,1 4594. 6 Aug. 2022, doi:10.1038/s41467-022-32283, GSE149614), which contained paired tumor and paracancerous tissue samples from 10 untreated primary HCC patients. In the original experiment, fresh tumor tissue (>1 cm^3^) was digested for 10× Genomics 3’ scRNA-seq library preparation, with ~5,000 target cells per library. Sequencing was performed on Illumina NovaSeq with ≥180 G raw data per sample. Residual tumor and paracancer tissues were snap-frozen at −80 °C for subsequent staining validation. Library construction strictly followed official GemCode microfluidic protocols for single-cell capture, reverse transcription, cDNA amplification and adapter ligation.

### 5.2 Raw sequencing data preprocessing

Raw FASTQ reads were processed with Cell Ranger v2.2.0 using the ‘cellranger count’ pipeline. Reads were aligned to human reference genome GRCh38, followed by UMI deduplication and gene expression quantification to generate sparse expression matrices.

### 5.3 Single-cell data quality control and normalization

All single-cell analyses were performed in R v3.6.0 and Seurat v4.0 (https://satijalab.org/seurat) following official tutorials. Low-quality cells were filtered: detected genes <500 or >8000, mitochondrial gene ratio >15%, and genes expressed in <3 cells were excluded. Expression normalization used the default LogNormalize algorithm (scale factor=10,000, log transformation).

### 5.4 Dimensional reduction, clustering and B cell annotation

Top 1000 highly variable genes per sample were intersected as integration features via ‘FindVariableFeatures’. ‘RunMultiCCA’ and ‘AlignSubspace’ were used for batch correction; the top 10 aligned components were input into SNN clustering (resolution = 0.3) to stratify B lineage cells into five subsets, including the C2 plasma cell subpopulation. PCA and t-SNE were used for visualization. Canonical lineage markers annotated all cell populations. ‘FindMarkers’ screened subset signature genes; GSVA calculated pathway activity scores. Bulk deconvolution based on B cell signatures was conducted on TCGA-LIHC dataset to estimate subset infiltration abundance.

### 5.5 Quantification of plasma cell exhaustion score based on cytokine loss signatures

A cytokine loss-associated exhaustion gene panel (*PDCD1*, *FCRL4*, *FCRL5*, *TBX21*, *ITGAX*, *TOX*) was used to calculate single-cell plasma cell exhaustion module scores with Seurat’s AddModuleScore function. Tumor-localized B lineage cells were isolated and split into two groups: C2 plasma cell subset and other tumor B cell subsets. Only genes detected in the filtered cell dataset were retained for module score calculation. Wilcoxon rank-sum tests were used to compare exhaustion scores between groups, and mean ± standard error bar charts with statistical annotations were generated via ggpubr and ggplot2.

### 5.6 Single-cell cytokine expression visualization

To compare the expression levels of key immunosuppressive cytokines across all B-cell subclusters, we extracted normalized RNA expression values of *IL10*, *IL-35* and *TGFB*. Cluster identity was relabeled from original clustering IDs to standardized C1–C5 nomenclature as a categorical factor. We used the ggpubr package to generate mean ± standard error bar charts for each cytokine. Each subcluster (C1–C5) was assigned a fixed distinct color for consistent visualization. For each target gene, the y-axis range was automatically scaled to 112% of the maximum expression value of that gene to avoid truncation of error bars; if all expression values equaled zero, the upper y-limit was set to 0.1.

### 5.7 Differential expression analysis of C2 plasma cells

The C2 plasma cell subpopulation was isolated from total B lineage cells. Cells were grouped by tissue origin (tumor vs. paracancer tissue). The Wilcoxon rank-sum test implemented in ‘FindMarkers’ was adopted for differential gene screening with thresholds log2FC > 0.25 and min.pct = 0.1; p-values were corrected via Bonferroni adjustment. Genes with significantly altered expression were sorted by log2 fold change for volcano plot visualization. *FTH1* was identified as a markedly upregulated gene in tumor-infiltrating C2 plasma cells.

### 5.8 Single-cell statistical methods

Statistical analyses were performed in SPSS 25.0. Normal data: mean ± SD; non-normal data: median (IQR). Two groups: t-test/Wilcoxon test; categorical variables: Chi-square/Fisher’s exact test (n<5). Multiple groups: one-way ANOVA with LSD-t and SNK *post-hoc* tests. Two-tailed P < 0.05 indicated statistical significance. Fluorescence and confocal microscopes captured cellular images.

### 5.9 Immunohistochemistry (TMA)

A 180-spot FFPE HCC tissue microarray (Lot XT16-029, Shanghai Outdo Biotech) was purchased. This TMA cohort, 102-case surgical clinical cohort and TCGA-LIHC dataset were mutually independent without overlapping samples. All specimens were ethically reviewed by the vendor in compliance with the revised Declaration of Helsinki (2013); no extra institutional ethics approval was required as only commercial finished chips were used. Slides were pre-fabricated 3 μm paraffin sections; standard dewaxing, rehydration and IHC staining were performed *in vitro*.

### 5.10 Multiplex immunofluorescence (Opal TSA)

Three-plex Opal TSA staining targeting *FTH1*, plasma cell marker and CK was conducted on the above TMA, with Opal 520/620/690 fluorophores (dilution = 1:100). AR6 buffer was used for heat-induced antigen retrieval in each cycle. Slides were baked, dewaxed and blocked before sequential primary antibody incubation (*FTH1* 1:25000; plasma cell marker 1:15000; CK 1:5) and TSA signal amplification. Microwave stripping removed bound antibodies while retaining fluorophores after each round. DAPI counterstaining and anti-fade mounting were completed, followed by multispectral imaging and spectral unmixing for quantitative analysis.

### 5.11 Clinical cohort and nomogram construction

#### 5.11.1 Cohort and ethics

A total of 102 radical resection HCC patients (Mar 2020–Mar 2022, PLA Medical School Hepatobiliary Surgery) formed an independent retrospective cohort, separate from TMA and TCGA samples. All patients provided written informed consent. The study was approved by the institutional ethics committee (S2018-111-01) and complied with the revised Declaration of Helsinki. Inclusion criteria: radical en-bloc resection, no preoperative distant metastasis, pathologically confirmed primary HCC, complete clinical and follow-up data with informed consent. Exclusion criteria: HCV/alcoholic liver disease, patients receiving preoperative or postoperative chemo/radiotherapy/immunotherapy, lost-to-follow-up subjects. Baseline information: 67 males and 35 females, mean age 53.89 ± 8.2 years; 43 patients with smoking history and 45 patients with diabetes mellitus.

### 5.12 Collected variables

Preoperative indicators: age, gender, smoking/diabetes history, NLR, ALT, AFP, GGT, TBIL, ALP. Postoperative indicators: *FTH1* expression level, Ki-67 index, tumor size, tumor multiplicity, differentiation grade, macrovascular invasion, operation duration.

### 5.13 Follow-up

Follow-up schedule: re-examination at 1 and 3 months after surgery, quarterly within 2 years, semi-annually after 2 years. Endpoints: tumor recurrence, all-cause death. Re-examination items included blood tests and contrast-enhanced chest/abdominal CT scans.

### 5.14 Statistical analysis and nomogram construction

X-tile software was applied to calculate the optimal cut-off value of NLR. Kaplan-Meier survival curves combined with Log-rank test were used to compare overall survival (OS) between subgroups. Univariate and multivariate Cox proportional hazards regression models were sequentially performed to screen independent prognostic factors. Statistically significant predictors from univariate Cox analysis were integrated via the R ‘rms’ package to construct OS nomogram. Model discrimination and calibration were validated by bootstrap-corrected C-index, time-dependent ROC curves and calibration curves (R packages: ‘rms’, ‘timeROC’). For statistical comparison, normal continuous variables were expressed as mean ± SD and analyzed by t-test; non-normal continuous variables were presented as median (IQR) and analyzed by Mann-Whitney U test; categorical variables were compared by Chi-square test or Fisher’s exact test. Two-tailed P < 0.05 was statistically significant. All statistical analyses were conducted using SPSS 25.0 and R v3.6.0.

## Data Availability

The original contributions presented in the study are included in the article/**Supplementary Material**. Further inquiries can be directed to the corresponding authors.

## Supplementary material

Supplementary Figure 1 Phenotypic characteristics of the C2 plasma cell subset. Violin plots showing the expression distribution of canonical B-cell and plasma cell markers in the C2 subset. The C2 plasma cells exhibited negligible expression of mature B-cell markers CD19 and CD20, low to moderate expression of the plasma cell transcription factor PRDM1, and high expression of the mature plasma cell markers CD138 and MZB1.

Supplementary Figure 2 Expression of immunosuppressive cytokines across five B-cell subsets. Bar plots showing the mean expression levels (± standard error) of immunosuppressive cytokines IL-10, IL-35, and TGF-β in the five distinct B-cell populations (C1–C5). The C2 plasma cell subset displayed significantly lower expression of all three immunosuppressive cytokines compared with other B-cell subsets.

Supplementary Figure 3 Exhaustion module scores across different B-cell subsets in HCC lesions. (Left) Bar plot comparing the mean exhaustion module score (± standard error) between the C2 plasma cell subset and all other tumor-infiltrating B-cell subsets. The C2 subset exhibited a significantly higher exhaustion score than other B-cell populations (***, P < 0.001). (Right) Violin plot showing the distribution of exhaustion module scores across all five B-cell subsets (C1–C5), with significant differences detected by the Kruskal-Wallis test (P < 2.2e-16).

Supplementary Figure 4 Transcriptional profiles of immune-regulatory, iron metabolism, and ferroptosis-related genes in C2 plasma cells across different tissue compartments. **(A)** Heatmap showing the expression of immune regulatory genes, including antigen-presenting molecules, co-stimulatory and co-inhibitory receptors, cytokines, and transcription factors. **(B)** Heatmap of core iron metabolism regulators. **(C)** Heatmap of canonical ferroptosis-related genes. All three heatmaps include C2 plasma cells isolated from lymph node (Lymph), paracancerous normal liver (Normal), portal vein tumor thrombus (PVTT), and primary HCC lesions (Tumor. Red indicates high gene expression, and blue indicates low expression.

Supplementary Figure 5 Dot plot of core genes from three functional submodules of SPERMATOGENESIS in C2 tumor plasma cells and normal plasma cells. Dot size = percentage of gene-expressing cells; color = average normalized expression. Germ cell markers are barely expressed in both groups, while iron metabolism genes *FTH1* and *FTL* are specifically elevated in C2 cells.
